# Effect of intensity modulated radiotherapy on lymphocytes in patients with esophageal squamous cell carcinoma and its clinical significance

**DOI:** 10.3389/fonc.2023.1096386

**Published:** 2023-03-07

**Authors:** Xiufang Tian, Yong Hou, Jianping Guo, Haiyan Wu, Limin Nie, Hang Wang, Yan Zhang, Yajuan Lv

**Affiliations:** ^1^ Department of Oncology, The First Affiliated Hospital of Shandong First Medical University & Shandong Provincial Qianfoshan Hospital, Shandong Key Laboratory of Rheumatic Disease and Translational Medicine, Shandong Lung Cancer Institute, Jinan, Shandong, China; ^2^ Department of Oncology, Maternal and Child Health Care Hospital of Zibo, Zibo, Shandong, China; ^3^ Department of Pharmacy, Central Hospital Affiliated to Shandong First Medical University, Jinan, Shandong, China; ^4^ Department of Pathology, Caoxian People's Hospital, Shandong, Heze, China; ^5^ Department of Graduate, Shandong First Medical University, Jinan, Shandong, China; ^6^ Department of Clinical Medicine, Shandong University, Jinan, Shandong, China

**Keywords:** esophageal squamous cell carcinoma, lymphopenia, nutritional factors, survival, radiotherapy

## Abstract

**Background:**

Radiotherapy usually leads to a decrease in the total number of lymphocytes in patients with esophageal cancer. The factors that causing lymphopenia and the clinical significance of lymphopenia are studied in this article.

**Patients and methods:**

110 patients with esophageal squamous cell carcinoma who had undergo intensity-modulated radiation therapy were enrolled. Statistical methods were used to analyze the correlation between lymphopenia and total survival in patients with esophageal cancer during radiotherapy, and analyze the correlations between nutritional factors and lymphopenia.

**Results:**

There were 11 patients with the lowest lymphocyte value with level 1-2 during radiotherapy, accounting for 10% of all the patients, and 110 patients with level 3-4, accounting for 90% of all the patient. In all the enrolled patients, the incidence of lymphocyte nadir G1, G2, G3 and G4 MinALC during radiotherapy accounted for 0.91%, 9.09%, 62.73% and 27.27%, respectively.KM survival analysis showed that the overall survival of patients in the group (MinALC ≤ 0.41×10^9^/L) was significantly lower than that of the patients in the other group (MinALC>0.43×10^9^/L). Nutritional indicators were positively correlated with the decline degree of lymphocytes. The minimal value of lymphocyte can predict the occurrence of grade 3-4 radiation pneumonitis.

**Conclusion:**

Lymphopenia induced by radiotherapy can predict survival and radiation pneumonitis. Nutritional factors such as hemoglobin and albumin were positively correlated with total lymphocytes numbers induced by radiotherapy.

## 1 Introduction

Esophageal cancer is a malignancy that has a poor prognosis, which is mainly due to patients presenting once the cancer is in the advances stages. Esophageal cancer(EC) is a serious malignancy with a poor prognosis (1). It has been reported 5-year survival rate of about 20% in patients with esophageal cancer (2). In the world,EC is the eighth most common cancer, and the sixth deadliest (3, 4). There are two main reasons for the poor prognosis of esophageal cancer, one is diagnosis at advanced stages, and the other is that it is prone to distant metastasis (1). Concurrent chemoradiotherapy is the main treatment for locally advanced esophageal cancer. Human immune function plays an important role in tumor appearance, development, and prognosis. Radiotherapy can activate the immune system by generating an inflammatory response, inducing cytokine signaling cascades or promoting the release of tumor antigens or directly killing tumor cells to reduce tumor burden (5). In addition, the radiation dose and the duration of radiotherapy will damage the lymphocytes circulating in the radiation field to varying degrees, thereby inhibiting the immune function of the human body.

Treatment-related lymphopenia is closely associated with the prognosis of many malignancies, including esophageal cancer (6). In patients with esophageal cancer, it has been reported that lymphopenia during chemoradiotherapy is associated with worse progression-free survival (PFS) and overall survival (OS) (7).

The main purpose of this study was to investigate the changing trend of the total number of peripheral blood lymphocytes (ALC) in different periods of Intensity-Modulated Radiation therapy(IMRT) exposure and its clinical significance. We hypothesized that lymphocytes reduction is related to the overall survival of patients, and studied the correlation between the degree of lymphocyte reduction caused by radiotherapy and overall survival. This study also assumes that there is a correlation between the nutritional status and the degree of lymphocyte reduction. The most commonly used nutritional indicators such as albumin and hemoglobin were studied in this study.

## 2 Criteria for patient selection

All esophageal cancer patients enrolled in this study were from the radiotherapy department of our hospital. All esophageal cancer patients must meet the following criteria (1): Histopathologically diagnosed ESCC; (2) No surgery and no any anti-tumor therapy such as radiochemotherapy or immune, targeted therapy; (3) ECOG score of 0-2. (4) Age≥18 years old. (5) Before the start of radiotherapy, the liver function and renal function tests were less than 2 times of the upper limit of the normal range. The exclusion criteria are as follows: (1) a history of other primary malignancies other than esophageal cancer before radiotherapy;(2) serious medical diseases that may be life-threatening at any time, such as myocardial infarction, AIDS, uremia, etc. (3) Combined infectious diseases, rheumatism or blood system diseases. (4) Hypersplenism or splenectomy or liver resection or a history of organ transplantation. (5) Combined with distant organ metastasis. (6) Esophageal fistula or gastrointestinal bleeding occurred before treatment. (7) History of interstitial pneumonia or other pneumonia combined with abnormal pulmonary function.

All enrolled patients were clinically staged according to the American Joint Committee on Cancer/International Union Against Cancer(AJCC/UICC) esophageal cancer staging seventh edition. Between February 2013 and May 2020, a total of 110 patients with esophageal squamous cell carcinoma were enrolled. NCCN Guidelines (2022, version 1) recommended that radiotherapy and chemotherapy was the first choice for esophageal cancer patients with clinical stages of T1b-cT2,N+ or cT3-cT4b,N±. According to the clinical stage of AJCC(version 7), some patients in stage II,all patients in stage III and IVA are recommended to receive comprehensive treatment based on radiotherapy and chemotherapy. In our study,2 patients with stage I and 4 patients with stage IIA(cT2N0M0,G2-3) were enrolled. But these 6 patients refused surgery due to advanced age and other factors. According to the NCCN guidelines, patients with early stage esophageal cancer who refused surgery were recommended to radiotherapy. The remaining 104 patients with esophageal cancer were locally advanced stage, and radiotherapy ± chemotherapy were first recommended according to the NCCN guidelines. The radiotherapy indications of all the enrolled patients were reasonable. These patients were treated with intensity-modulated radiation therapy with or without concurrent chemotherapy in our hospital. The chemotherapy regimens for concurrent radiotherapy mainly include (1) platinum-based chemotherapy, cisplatin or nedaplatin (25mg/m^2^ a day) with or without docetaxel (25mg/m^2^ a day), once a week. (2) Oral fluorouracil chemotherapy drugs, capecitabine (850-1000mg/m^2^, twice a day) or Sigio capsules (50-60 mg/m^2^, twice a day) with concurrent radiotherapy orally every day.(3)Docetaxel (75mg/m^2^, one day), with or without cisplatin 25mg/m^2^ (days 1-3), once every 3 weeks.

## 3 General information and blood data collection and recording

The general information was collected through the hospital’s electronic medical record system, such as age, gender and so on. The nutritional status of patients is expressed by serum albumin, prealbumin and hemoglobin. Within 2 weeks before radiotherapy, during radiotherapy, and within 1 month after radiotherapy, the peripheral blood data of all enrolled esophageal cancer patients were recorded, including the total number of peripheral blood lymphocytes, hemoglobin, and albumin values, etc. The time point before radiotherapy is represented by T1, the time point after radiotherapy is represented by T2, and the minimum value of ALC during radiotherapy is considered to be the lowest point of lymphocytes, which is represented by MinALC. According to the standardized Common Criteria for Adverse Events(CTCAE5.0), the range of grade 1 ALC reduction was: 0.8×10^9^/L-lower limit of normal value, and the range of grade 2 ALC reduction was: 0.5-0.8×10^9^/L, the range of grade 3 ALC reduction was 0.2-0.5×10^9^/L, and the range of grade 4 ALC reduction was: 0-0.2×10^9^/L.Hypoalbuminemia was defined as serum albumin levels below 35 g/L.

## 4 Radiation therapy plan

All enrolled patients with esophageal cancer underwent treatment planning and dose distribution calculations using the Eclipse 10 planning system (Varian Medical Systems, Palo Alto, Calif). Large aperture CT (GE, discovery) was used for positioning, and head, neck and shoulder membranes or negative pressure pads were used for positioning and fixation. All patients were given IMRT. The radiotherapy prescription plan target volume (PTV) dose of the enrolled patients was 41.40-69.96 Gray(Gray, Gy)(once a day, 1.8-2.0 Gy each time, 5 times/week), and the median PTV prescription dose was 60Gy. All patients were irradiated with linear accelerator (truebeam,varian) X-rays. All radiotherapy plans are in accordance with the “National Comprehensive Cancer Network 2019 Guidelines for Esophageal Cancer”, all radiotherapy plans involved organs at risk must meet the organ dose-volume constraints.

## 5 Statistical analysis

All statistical analyses of data in this study were performed using SPSS 22.0 software (IBM SPSS, USA) and Medcalc software. Among them, parameters conforming to continuous normal distribution were expressed as mean ± standard deviation (SD) or median (minimum-maximum value). Independent samples t or chi-square tests were used to analyze the differences between continuous and categorical variables between groups. Paired samples t-test was used to compare the same parameters at different time points. The predictive significance of parameters for overall survival was analyzed by univariate COX regression analysis, where univariate COX analysis (P<0.1) was used to construct a multivariate risk survival model for COX multivariate analysis (forward: wald method). Receiver operating characteristic (ROC) curve analysis was used to evaluate the sensitivity and specificity of blood parameters in predicting overall survival (OS) with death status as the endpoint, and to determine cutoff points. The possible related factors of lymphocyte reduction were analyzed by ROC curve. Kaplan-Meier survival analysis was used to analyse the significance of individual parameters for predicting OS. Pearson analysis and scatter plot were used to analyze the correlation between nutritional parameters and MinALC/T1ALC ratio. All P values in this study were two-sided, and statistical significance was defined as P< 0.05.

## 6 Result

### 6.1 General characteristics of patients

Finally, a total of 110 patients with esophageal cancer in this study met the inclusion criteria and were included in this study. The general characteristics of patients with esophageal cancer were showed in **Table 1**. A total of 26 women and 84 men were enrolled in this study, and the median age at diagnosis of esophageal cancer was 67.5 years(range 36-79 years). Among them, there were 48 patients with cervical and upper thoracic esophageal cancer, 43 with middle thoracic esophageal cancer, and 19 with lower thoracic esophageal cancer. Among them, there were 2 patients with clinical stage I, 42 patients with stage II, 46 patients with stage III, and 20 patients with stage IV. There were 57 patients who received concurrent chemoradiotherapy and 53 patients who received only radiotherapy. A total of 24 patients had lymphopenia before radiotherapy, accounting for 21.82% of the total patients.The median prescribed dose of PTV was 6000cGy, ranging from (4140-6996)cGy. There were 11 patients with the lowest lymphocyte value with level 1-2 during radiotherapy, accounting for 10% of all the patients, and 110 patients with level 3-4, accounting for 90% of all the patients (**Table 1**). In all enrolled patients, the incidence of lymphocyte nadir G1, G2, G3 and G4 MinALC during radiotherapy accounted for 0.91%, 9.09%, 62.73% and 27.27%, respectively.

**Table 1:** Characteristics of patients with esophagus cancer.

Characteristic	Median(range)	Numbers of patients
Age (years)	67.5 (36-79)	
≤60		25
>60		85
Sex		
Male		84
Female		26
Tumor location		
Cervical or Upper thoracic		48
Middle thoracic		43
Lower thoracic		19
AJCC clinical stage		5
I		2
II		42
III		46
IVA		20
Radiation dose(cGy)	6000 (4140-6996)	
Concurrent chemotherapy		
No		53
Yes		57
Pre RT lymphopenia		
Yes		24
No		86
MinALC during RT		
Grade1-2		11
Grade3-4		99

### 6.2 Comparison of peripheral blood parameters in single radiotherapy and concurrent chemoradiotherapy groups

The number of white blood cells in the single radiotherapy group was slightly higher than that in the concurrent chemoradiotherapy group before RT(P=0.049, **Table 2**). During radiotherapy, there was no significant difference in the nadir value of lymphocytes between the two groups, and the white blood cells in the concurrent chemoradiotherapy group were significantly lower than those in the single radiotherapy group (P=0.012, **Table 2**). There was no significant difference in the total number of white blood cells and lymphocytes between the two groups in 1 month after radiotherapy (**Table 2**).

**Table 2:** Comparison of WBC and ALC in patients with or without concurrent chemotherapy at different time points.

Parameters	Single RT	CRT	t	P
WBC(T1)	7.25 ± 3.03	6.28 ± 1.90	1.994	0.049
ALC(T1)	1.59 ± 0.54	1.60 ± 0.76	-0.135	0.893
Min WBC	3.43 ± 1.04	2.96 ± 0.92	2.555	0.012
Min ALC	0.32 ± 0.16	0.28 ± 0.14	1.43	0.155
WBC(T2)	5.17 ± 3.17	4.88 ± 2.96	0.499	0.618
ALC(T2)	0.46 ± 0.28	0.47 ± 0.33	-0.224	0.824

### 6.3 Dynamic changes of peripheral blood parameters at different time points of radiotherapy

White blood cells, total lymphocytes, hemoglobin and albumin, and serum prealbumin were significantly decreased during radiotherapy compared with those before radiotherapy (**Table 3**,P<0.001). All peripheral blood parameters after RT were still significantly lower than those before RT other than albumin and prealbumin. (P<0.001, **Table 3**).

**Table 3:** Dynamic change of parameters in esophageal patients over time.

Parameters	T1	Min value during RT	T2	Total P	P1	P2	P3
WBC	6.75 ± 2.55	3.19 ± 1.01	5.02 ± 3.05	<0.001	<0.001	<0.001	<0.001
ALC	1.60 ± 0.66	0.30 ± 0.15	0.47 ± 0.30	<0.001	<0.001	<0.001	<0.001
Hemoglobin	129.99 ± 17.46	113.12 ± 14.74	16.04 ± 17.89	<0.001	<0.001	0.037	<0.001
Albumin	40.29 ± 5.03	36.57 ± 4.80	36.79 ± 5.62	<0.001	<0.001	0.587	<0.001
Prealbumin	208.94 ± 58.36	179.79 ± 61.54	180.38 ± 61.15	<0.001	<0.001	0.908	<0.001

Total P, comparison between the three groups; P1, comparison between T1 and Min value during RT; P2, comparison between T1 and T2; P3, comparison between Min value during RT and T2.

### 6.4 KM survival analysis of lymphocyte count and overall survival

**Figure 1:**
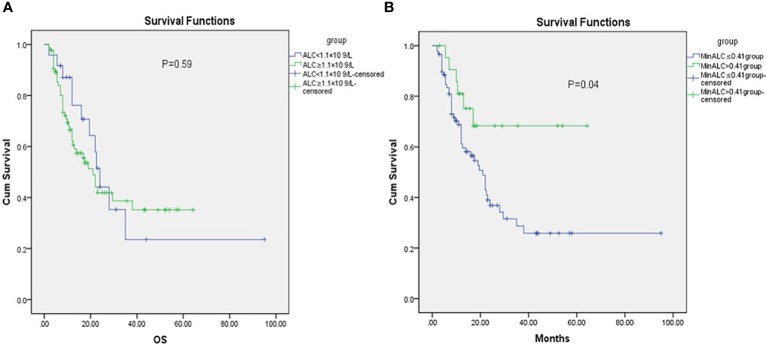
KM analysis of OS before RT [**(A)** ALC<1.1×10^9^/L VS ALC≥1.1×10^9^/L,P=0.59, **(B)** MinALC>0.41×10^9^/L VS MinALC≤0.41×10^9^/L,P=0.04].

Lymphopenia before RT was defined as ALC<1.1×10^9^/L. Before radiotherapy, whether the peripheral blood lymphocyte count was less than 1.1×10^9^/L was divided into two groups. There was no significant difference between the two groups in KM survival analysis (**Figure 1A**, P=0.59). According to the end point of death or not, ROC curve analysis found the cut-off value of the lowest lymphocyte value during radiotherapy (MinALC ≤ 0.41×10^9^/L) and divided all patients into two groups. KM survival analysis was used to compare the survival difference between these two groups. The overall survival of patients in the group (MinALC ≤ 0.41×10^9^/L) was significantly lower than that of the patients in the other group (MinALC>0.43×109/L, **Figure 1B**, P=0.04).

### 6.5 Univariate, multivariate COX overall survival analysis

Univariate COX analysis was performed on each parameter that might be predict survival, and then a multivariate COX proportional hazards survival model was constructed. Univariate COX test showed that the parameter that may predict the overall survival were clinical stage, MinALC(≤0.41 vs >0.41) (**Table 4**, P<0.05). Univariate COX analysis (P<0.1) entered into COX multivariate analysis (forward:wald). In addition to the above 2 factors, MinPA (<200 vs. ≥200mg/L) also entered in multivariate regression analysis. Finally, COX multivariate analysis showed that MinALC may predict the overall survival (see **Table 4**, P<0.05).

**Table 4:** Univariate and multivariate COX analysis of overall survival for all patients.

Parameters	Univariate			Multivariate		
	HR	95% CI	P	HR	95% CI	P
Age (≤60 vs>60)	1.209	0.743-1.967	0.445			
Sex (male vs female)	1.069	0.658-1.736	0.787			
Stage (I-II vs III-IV)	1.359	1.009-1.829	0.043			
T1WBC (>8.24 vs ≤8.24)	0.503	0.756-1.771	0.503			
T1ALC (≤1.1vs>1.1)	0.94	0.586-1.507	0.796			
MinALC (≤0.41 vs >0.41)	0.032	0.004-0.264	0.001	0.032	0.004-0.264	0.001
CRT vs single RT	0.744	0.484-1.144	0.178			
MinAlb (<35 vs ≥35g/L)	0.748	0.483-1.161	0.748			
MinPA (<200vs ≥200mg/L)	0.662	0.426-1.030	0.067			

### 6.6 Correlation between the ratio of minimal ALC to ALC before RT and nutritional factors

**Figure 2:**
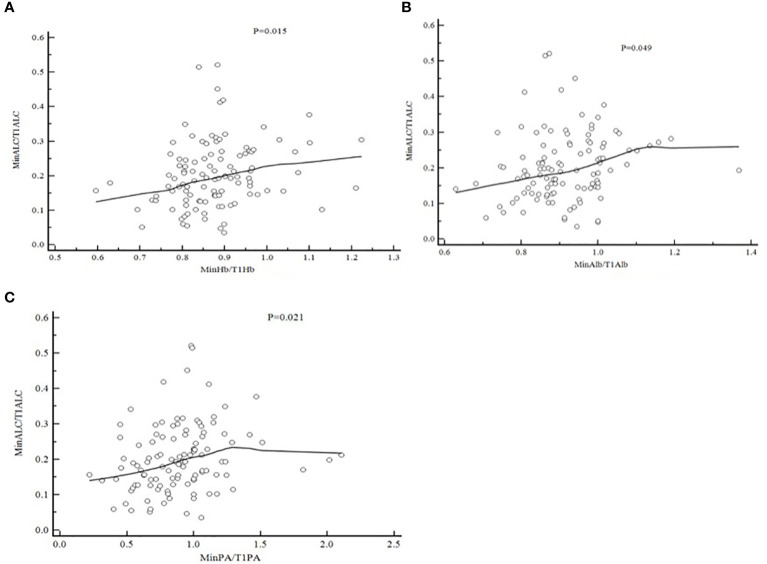
Pearson Correlation of MinALC/T1ALC and nutritional factors [**(A)** MinHb/T1Hb and MinALC/T1ALC,P=0.015; **(B)** MinA1b/T1A1b and MinALC/T1ALC,P=0.049; **(C)** MinPA/T1PA and MinALC/T1ALC,P=0.021].

In order to find whether there is a correlation between the degree of lymphocyte reduction during radiotherapy and nutritional factors, we take MinHb/T1Hb, MinAlb/T1Alb, MinPA/T1PA as the X-axis, and MinALC/T1ALC as the Y-axis.Pearson correlation bivariate statistical analysis was used. The results were shown in **Figure 2**, MinHb/T1Hb as the X-axis,P=0.015,MinAlb/T1Alb as the X-axis,P=0.049,MinPA/t1PA as the X-axis,P=0.021.These three nutritional indicators were positively correlated with the decline degree of lymphocytes.

### 6.7 ROC curve predicts the occurrence of grade 3-4 radiation esophagitis and radiation pneumonitis

**Figure 3:**
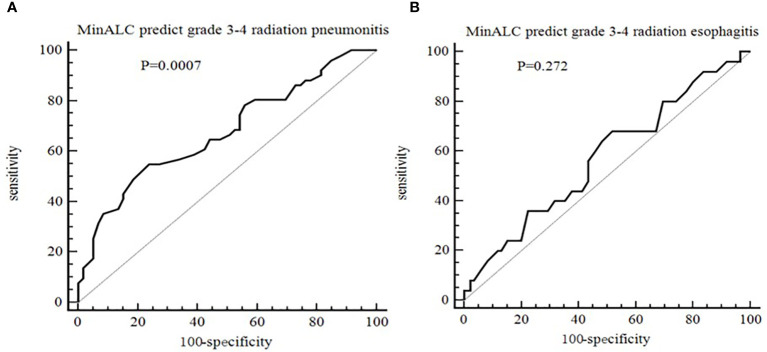
ROC curve for the prediction of radiation side effect [**(A)** MinALC predict grade 3-4 radiation pneumonitis, AUC=0.676,P=0.0007; **(B)** MinALC predict grade 3-4 radiation esophagitis, AUC=0.573,P=0.272].

As shown in **Figure 3**, taking the occurrence of grade 3-4 radiation esophagitis and radiation pneumonitis as the end points, and the MinALC during radiotherapy as a variable, which is calculated by ROC curve analysis. The results showed that MinALC predicted the occurrence of grade 3-4 radiation pneumonitis (AUC=0.676,P=0.0007, **Figure 3A**). However, the prediction of the occurrence of grade 3-4 radiation esophagitis was poor (AUC=0.573,P=0.272, **Figure 3B**).

### 6.8 Pearson correlation of MinALC/T1ALC and radiation dose factors

In order to study the correlation between dose and units which related to the degree of lymphopenia, we collected and recorded dosimetric parameters through DVHs. We take MinALC/T1ALC as the Y-axis, and physical factors such as the average dose of PTV, Mus as X-axis, and Pearson bivariate analysis was used to analysis. The degree of MinALC/T1ALC reduction is significantly negatively correlated with the following dose factors(heart max dose, heart mean dose and lung mean dose)(P<0.05, **Table 5**).

**Table 5:** Pearson correlation of MinALC/T1ALC and radiation dose factors.

Parameters	MinALC/T1TLC		
	r	95%CI	P
PTV max dose	-0.046	-0.232−0.141	0.626
PTV mean dose	-0.032	-0.218−0.156	0.741
Heart max dose	-0.209	-0.381−-0.023	0.028
Heart mean dose	-0.268	-0.433−0.085	0.005
Lung max dose	-0.071	-0.255−0.118	0.459
Lung mean dose	-0.348	-0.502−-0.172	<0.001
Machine Units	-0.129	-0.309−0.059	0.177

## 7 Discussion

### 7.1 The relationship between lymphopenia induced by radiotherapy and prognosis

Esophageal cancer has the characteristics of high incidence and high mortality, and has an extremely poor prognosis, with a median 5-year survival rate of about 15%-25% (8). Peripheral blood lymphocytes are one of the important members of the human immune system and can reflect the immune function of the human body. Radiation therapy induces lymphopenia, which is associated with a radiation-induced immunosuppressive effect and is common in patients with malignancies (9, 10). One study had reported that grade 4 lymphopenia during chemoradiation was occurred in approximately 31 percent of all the patients (9). Studies have shown that about 37% of patients have grade 4 lymphopenia during CRT, and patients with grade 3-4 lymphopenia are more likely, about 91% (10). This study also reported that patients with grade 4 lymphopenia during CRT had significantly shorter PFS and OS than those without grade 4 lymphopenia (PFS: median 19.1 months vs. 61.7 months and OS: median 34.7 and 63.1 months, respectively) (10). A study had reported that radiotherapy combined with or without chemotherapy can induce severe lymphopenia, which is closely related to the prognosis of patients with various malignant tumors (7). The authors of Davuluri et al. found that the probability of grade 1, 2, 3, and 4 lymphopenia during CRT for esophageal cancer was 2%, 12%, 59%, and 27%, respectively. However, only grade 4 lymphopenia was associated with worse OS (7). An article found that patients with grade 4 lymphopenia had a poorer clinical prognosis, including OS, PFS, and distant metastasis-free survival than patients without grade 4 lymphopenia (11). A study of radiotherapy for esophageal cancer showed that the tumor progression rate and cancer-related mortality were significantly higher in the post-treatment lymphopenia group than in the post-treatment ALC count≥200 cells mm^3^ group (76.4% vs. 52.8%, P<0.001; 58.4% vs. 39.6%,P=0.003) (9). The mechanisms underlying the association of lymphopenia with poorer survival in malignancies are not fully understood. Animal experiments have found that radiation can promote the release of antigens to stimulate lymphocytes, which can activate lymphocyte-mediated anti-tumor immune responses, resulting in anti-tumor effects (12). Some authors have found that tumor-infiltrating lymphocytes increase in patients after radiotherapy and chemotherapy, which can recognize non-natural antigens and then lead to tumor cell death (13). The correlation between lymphocyte reduction and prognosis may be related to the inhibition of immune function by radiotherapy. First, radiotherapy rays that cause suppression of bone marrow activity which leading to a decrease in the number of lymphocytes generated. And rays can also damage lymphoid organs such as the thymus or spleen, which may lead to immunosuppression (14). In addition, radiotherapy rays can directly damage the normal function of peripheral blood lymphocytes and directly inhibit immune function (9). Our study found that there was no significant difference in the nadir value of lymphocytes in patients with or without combined chemotherapy during radiotherapy (**Table 2**). In addition, WBC, hemoglobin, albumin, and serum prealbumin were significantly decreased during radiotherapy compared with those before radiotherapy(**Table 3**, P<0.001). After radiotherapy, all parameters recovered,but ALC recovered more slowly (**Table 3**). The OS of patients in the MinALC ≤ 0.41×10^9^/L group was significantly lower than that of the patients in the MinALC>0.41×10^9^/L group (**Figure 2B**, P=0.04). In addition, COX multivariate analysis showed that MinALC(≤0.41 vs.>0.41×10^9^/L) was significantly associated with OS (**Table 4**, P=0.001).

### 7.2 The relationship between nutritional factors and lymphocytes

In addition to immunity status, the nutritional status of individuals is closely related to the prognosis of various malignancies (15, 16). The most typical clinical symptom of esophageal cancer patients is a progressive feeling of blocking eating. Because of direct involvement in eating, the probability of malnutrition in patients with esophageal cancer ranks first among all malignant tumors (17). Low albumin is a common malnutrition expression in patients with malignant tumors, which is a typical manifestation of cachexia and is related to the prognosis of patients with various malignant tumors, including esophageal cancer (18, 19). Many studies have shown that albumin can prevent tumorigenesis by stabilizing cell growth and inhibiting DNA replication (20). Basic experiments show that the activation of T and B lymphocytes *in vitro* requires the presence of serum albumin (21). High-protein dietary intervention in cancer patients may stimulate the body’s immune response.A study had showed that a low-carbohydrate, high-protein combination diet (10.6% carbohydrate, 63.5% protein) slows down the rate of tumor growth in mice compared to a traditional diet (55.2% carbohydrate, 23.2% protein) (22). And mice with a high-protein diet had less chromosomal damage in the bone marrow and reduced oxidative damage in the liver and spleen compared with mice with a low-protein diet (23). A study found that a lower level of albumin was an independent predictor of early death (less than 6 months) in esophageal cancer (18). Lower albumin levels not only reflect poorer nutritional status, but also reflect tumor aggressiveness status (18). Low hemoglobin, or anemia, is the main determinant of whether human tumor cells are hypoxic and can directly affect the sensitivity of tumor cells to radiotherapy (24). Studies have shown that malnutrition such as anemia causes many adverse clinical consequences, including reduced sensitivity to treatments such as RT, increased risk of treatment toxicity during anti-tumor periods, and reduced survival (25). At present, whether there is a correlation between the changes of hemoglobin and albumin and the decrease of lymphocytes during radiotherapy for esophageal cancer has not been reported. Patients with cachexia usually experience lymphopenia due to decreased production of cell-stimulating factors (26). Our study showed that MinHb/T1Hb,MinAlb/T1Alb,MinPA/t1PA were significantly positively correlated with the ratio of MinALC/T1ALC(**Figure 2**, P<0.05). Our study showed that the nutritional status of patients is closely related to the degree of lymphopenia, so it is important to strengthen nutritional support for patients with esophageal cancer during radiotherapy.

### 7.3 The relationship between lymphopenia induced by radiotherapy and radiation pneumonitis

Zhou et al. showed that the decrease in lymphocyte count in lung cancer patients reflected the severity of radiation pneumonitis. Values of lymphocytes and CD4+ T lymphocyte subsets proved as independent predictors of radiation pneumonitis. The lower peripheral blood levels of lymphocytes and CD4+ T lymphocyte were associated with an increased risk of radiation pneumonitis, which was validated by this mice model (27). Yang et al. showed that the platelet-to-lymphocyte ratio during treatment (P=0.027), and neutrophil-to-lymphocyte ratio at the end of treatment (P=0.001) were the independent predictors for symptomatic radiation pneumonitis in patients with Esophageal Cancer (28). Zhang et al. revealed that Higher CD8+ T cell count after radiotherapy in lung cancer patients was associated with an increased risk of radiation pneumonitis (29). The neutrophil-lymphocyte ratio(NLR) was higher in patients who developed symptomatic radiation pneumonitis (p=0.012).The NLR is a useful biomarker for predicting symptomatic radiation pneumonitis development after RT in NSCLC patients (30). And a study showed that lymphocyte percentage was related to radiation pneumonia in patients with lung cancer after RT(P<0.05) (31). Recent a study showed that Pre- and post-RT percentage of CD8+ T cell were the independent factors of ≥grade 2 radiation pneumonia in patients with esophageal squamous cell carcinoma (32). In our study, we found that MinALC could predict the occurrence of grade 3-4 radiation pneumonitis (P=0.0007, **Figure 3A**). This result may be related to the decrease of lymphocytes that caused by radiation rays, which leads to a decline in immune function and is more likely to cause pneumonia. Due to the limited sample size, this result still needs to be confirmed by a large sample of clinical trials. Severe pneumonia can directly lead to the death of patients. Therefore, for patients with poor lung function before radiotherapy, the irradiated volume and dose of bilateral lungs should be strictly controlled.

This study has its own limitations. Firstly, this study is a retrospective study, maybe there are many selective biases when selecting patients (for example, more elderly patients were enrolled, because elderly patients are more inclined to refuse surgery). Secondly, due to the small number of patients enrolled, the subgroup analysis is not conducted for different groups of chemotherapy schemes and chemotherapy doses. Thirdly, due to the limitation of the retrospective study, the factors that may cause lymphopenia cannot be fully included, such as whether the patient uses other drugs that may cause lymphopenia during radiotherapy. Therefore, all the findings of this article still need to be further confirmed by large-scale prospective research.

## 8 Conclusion

Although there were many limitations in this article, the results still showed that lymphopenia can be used to predict survival and radiation pneumonitis. The decline of total lymphocytes values during radiotherapy could predict the survival time of esophageal cancer. Nutritional factors such as hemoglobin and albumin were positively correlated with total lymphocytes values induced by radiotherapy. During radiotherapy, strengthening nutritional support may reduce the degrees of lymphopenia caused by radiotherapy and may prolong the survival time.

## Data availability statement

The raw data supporting the conclusions of this article will be made available by the authors, without undue reservation.

## Ethics statement

The studies involving human participants were reviewed and approved by Shandong Qianfoshan Hospital (Number:S008). Written informed consent for participation was not required for this study in accordance with the national legislation and the institutional requirements.

## Author contributions

(I) Conception and design: YL. (II) Collection and assembly of data: YH, XT, LN. (III) Data analysis and interpretation: JG, HWu, YZ, HWa. (IV) Manuscript writing: All authors. (V) All authors contributed to the article and approved the submitted version.
